# Mycoplasma genitalium Prevalence, Coinfection, and Macrolide Antibiotic Resistance Frequency in a Multicenter Clinical Study Cohort in the United States

**DOI:** 10.1128/JCM.01053-16

**Published:** 2016-08-24

**Authors:** Damon Getman, Alice Jiang, Meghan O'Donnell, Seth Cohen

**Affiliations:** a Hologic, Inc., San Diego, California, USA; b Occidental College, Los Angeles, California, USA; Mayo Clinic

## Abstract

The prevalence rates of Mycoplasma genitalium infections and coinfections with other sexually transmitted organisms and the frequency of a macrolide antibiotic resistance phenotype were determined in urogenital specimens collected from female and male subjects enrolled in a multicenter clinical study in the United States. Specimens from 946 subjects seeking care from seven geographically diverse clinical sites were tested for M. genitalium and for Chlamydia trachomatis, Neisseria gonorrhoeae, and Trichomonas vaginalis. Sequencing was used to assess macrolide antibiotic resistance among M. genitalium-positive subjects. M. genitalium prevalence rates were 16.1% for females and 17.2% for males. Significant risk factors for M. genitalium infections were black race, younger age, non-Hispanic ethnicity, and female symptomatic status. Female M. genitalium infections were significantly more prevalent than C. trachomatis and N. gonorrhoeae infections, while the M. genitalium infection rate in males was significantly higher than the N. gonorrhoeae and T. vaginalis infection rates. The macrolide-resistant phenotype was found in 50.8% of females and 42% of males. These results show a high prevalence of M. genitalium single infections, a lower prevalence of coinfections with other sexually transmitted organisms, and high rates of macrolide antibiotic resistance in a diverse sample of subjects seeking care across a wide geographic area of the United States.

## INTRODUCTION

Urogenital tract infections involving Chlamydia trachomatis and Neisseria gonorrhoeae have long been recognized as significant causes of adverse reproductive and sexual health outcomes. In the past decade, accumulating evidence has supported the hypothesis that sexual transmission of and infection with the mollicute Mycoplasma genitalium represent an additional cause of host inflammatory responses in female and male urogenital epithelia, being associated with significantly elevated risks of nongonococcal urethritis in men (1) and of cervicitis, pelvic inflammatory disease, preterm birth, infertility, and spontaneous abortion in women (2).

Due to the generally fastidious requirements for culturing of Mycoplasma species *in vitro*, molecular methods have been the predominant means for detecting M. genitalium in clinical specimens. The use of nucleic acid amplification tests (NAATs) employing PCR for detection of M. genitalium genomic DNA targets (3, 4) and transcription-mediated amplification (TMA) for detection of M. genitalium 16S rRNA (5–7) has increased our understanding of the epidemiology of M. genitalium, as well as associations between M. genitalium infections and reproductive health morbidities. Such studies revealed M. genitalium prevalence rates of approximately 1% in a screening population (8) and ranging from 9% to >50% in populations at high risk for sexually transmitted infections (STIs) (9–12). However, the cohorts evaluated were often of relatively small enrollment size, originated in narrow geographic locales in the United States, or represented subjects from homogeneous demographic groups (7, 12–16). In this study, we used a sensitive TMA-based NAAT to detect M. genitalium in specimens obtained from subjects enrolled in a multicenter clinical study encompassing a diverse sample of adolescent and adult women and men seeking care across a broad geographic area of the United States. We also determined the rates of coinfection of M. genitalium with C. trachomatis, N. gonorrhoeae, and Trichomonas vaginalis and assessed the rate of macrolide antibiotic resistance markers present in M. genitalium 23S rRNA isolated from a subset of the subjects studied.

(Some of the data included in this study were previously presented at the following conferences: 2014 National STD Prevention Conference, Atlanta, GA, 9 to 12 June 2014; 115th General Meeting of the American Society for Microbiology, New Orleans, LA, 30 May to 2 June 2015; International Union against Sexually Transmitted Infections-Europe Conference, Barcelona, Spain, 24 to 27 September 2015; and World STI & HIV Congress 2015, Brisbane, Australia, 17 to 20 September 2015.)

## MATERIALS AND METHODS

### Study population.

Specimens analyzed in this study were collected as part of a previous unpublished clinical study, for which participating subjects provided written informed consent. In brief, specimens were obtained from symptomatic (cervicitis, vaginitis, or urethritis for females or urethritis for males) and asymptomatic subjects enrolled from seven diverse U.S. clinical sites, including family medicine, obstetrics and gynecology (OB-GYN), family planning, public health, and sexually transmitted disease (STD) clinics. A total of 1,368 female subjects and 599 male subjects were enrolled between January 2013 and July 2014. For the purposes of the current analysis, only subjects with complete specimen collections and test results for M. genitalium, C. trachomatis, N. gonorrhoeae, and T. vaginalis were included. Thus, the final cohort used for the analysis consisted of 515 female subjects (14 to 70 years of age) and 431 male subjects (18 to 78 years of age). Demographic characteristics, enrollment sites, and the symptomatic status of the subjects are presented in Table 1. The majority of female and male subjects were of younger age (<30 yr), black race, and non-Hispanic ethnicity. The symptomatic status of each subject was determined by the attending physician at the time of the patient's visit to the health care facility.

**TABLE 1:** Prevalence of M. genitalium by subject age group, self-identified demographic status, and health care facility type

Category	No. with M. genitalium/total no. (% [95% CI])	
	Female	Male
Age		
14–17 yr	12/40 (30.0 [18.7–45.4])	No data
18–20 yr	35/140 (25.0 [18.6–32.8])	6/48 (12.5 [5.9–24.7])
21–30 yr	28/210 (13.3 [9.4–18.6])	53/220 (24.1 [18.9–30.2])
31–40 yr	7/73 (9.6 [4.7–18.5])	11/75 (14.7 [8.4–24.4])
41–50 yr	1/34 (2.9 [0.5–14.9])	3/49 (6.1 [2.1–16.5])
51–60 yr	1/16 (6.3 [1.1–28.3])	1/29 (3.4 [0.6–17.2])
61–70 yr	0/2 (0 [0–65.8])	0/9 (0 [0–29.9])
71–78 yr	No data	0/1 (0 [0–79.4])
≤30 yr	75/390 (19.2 [15.6–23.4])^*a*^	59/268 (22 [17.5–27.4])^*b*^
>30 yr	9/125 (7.2 [3.8–13.1])	15/163 (9.2 [5.7–14.6])
All females (14–70 yr)	83/515 (16.1 [13.2–19.6])	
All males (18–78 yr)		74/431 (17.2 [13.9–21])
Symptomatic status		
Symptomatic^*c*^	69/327 (21.1 [17–25.8])^*d*^	33/171 (19.3 [14.1–25.9])
Asymptomatic	14/188 (7.5 [4.5–12.1])	40/260 (15.4 [11.5–20.3])
Race/ethnicity		
Black	76/323 (23.5 [19.2–28.5])^*e*^	76/272 (27.9 [22.9–33.6])^*f*^
White	8/163 (4.9 [2.5–9.4])	5/117 (4.3 [1.8–9.6])
Asian	0/5 (0 [0–43.5])	0/2 (0 [0–65.8])
Unknown race	0/24 (0 [0–13.8])	4/40 (10.0 [4–23.1])
Hispanic	1/49 (2.0 [0.4–10.7])	4/75 (5.3 [2.1–12.9])
Non-Hispanic	77/401 (19.2 [15.6–23.3])^*g*^	63/294 (21.4 [17.1–26.5])^*h*^
Unknown ethnicity	6/65 (9.2 [4.3–18.7])	6/62 (9.7 [4.5–19.6])
Enrollment site		
Family medicine/OB-GYN, northeastern USA	2/84 (2.4 [0.6–8.3])	1/66 (1.5 [0.3–8.1])
Family planning clinic, southwestern USA	0/24 (0 [0–13.8])	14/127 (11.0 [6.7–17.7])
Public health clinic, southeastern USA	13/78 (16.7 [10–26.5])	35/153 (22.9 [16.9–30.2])
Public health clinic, mid-Atlantic USA	8/39 (20.5 [10.8–35.5])	20/79 (25.3 [17–35.9])
Hospital system high-risk STD clinics, midwestern USA	10/54 (18.5 [10.4–30.8])	3/6 (50.0 [18.8–81.2])
Adolescent gynecology clinic, midwestern USA	39/135 (28.9 [21.9–37])	No data
Family planning clinic, midwestern USA	4/22 (18.2 [7.3–38.5])	No data

a OR of 2.67 (*P* = 0.0075) versus females >30 years of age.

b OR of 2.39 (*P* = 0.0043) versus males >30 years of age.

c Clinician-diagnosed urethritis, vaginitis, or cervicitis in females or urethritis in males.

d OR of 2.83 (*P* < 0.0007) versus asymptomatic females.

e OR of 4.79 (*P* < 0.0001) versus white females.

f OR of 6.54 (*P* < 0.0001) versus white males.

g OR of 9.41 (*P* = 0.0276) versus Hispanic females.

h OR of 4.02 (*P* = 0.0089) versus Hispanic males.

### Laboratory procedures.

Four female specimens (clinician-collected vaginal swab, endocervical swab, ThinPrep Pap Preservcyt liquid [Hologic, Inc., San Diego, CA] cervical specimen, and self-collected urine specimen) and two male specimens (urethral swab and self-collected urine specimen) were obtained during single patient visits to the clinical sites. Swab and urine samples were collected into Aptima swab specimen and urine specimen transport tubes, respectively (Hologic), according to the manufacturer's package inserts and either were tested directly or were stored frozen at −70°C and tested at later dates.

Specimens were tested for M. genitalium using a research-use-only TMA assay for M. genitalium 16S rRNA, as described previously (5–7) except that testing was performed with the Tigris DTS system or the Panther system (both from Hologic), using a cutoff value of 100,000 relative light units. C. trachomatis, N. gonorrhoeae, and T. vaginalis testing was performed with the Panther system and the FDA-cleared Aptima Combo 2 and Aptima Trichomonas vaginalis assays (Hologic), according to the assay package inserts.

M. genitalium macrolide resistance was assessed using nested reverse transcription-PCR of M. genitalium 23S rRNA, using a modification of a previously described method (17), followed by Sanger sequencing. To detect mutations at base locations 2058 and 2059 (Escherichia coli numbering; located in region V of the 23S rRNA), which have been shown to be associated with M. genitalium macrolide resistance (18), M. genitalium rRNA was purified from clinical specimens using Aptima general purpose target capture reagent (Hologic) on a KingFisher purification system (Thermo Scientific). The reverse transcription and PCR primers used were as follows: reverse transcription, 5′-CCACCTAACACTGTCTTGAAACTGG-3′; first-round PCR, 5′-GGAGGTTAGCAATTTATTGCAAAGC-3′ (forward) and 5′-CCACCTAACACTGTCTTGAAACTGG-3′ (reverse); second-round PCR, 5′-CGTAACTATAACGGTCCTAAGGTAG-3′ (forward) and 5′-CACATCAACAAATCCTTGCGAAC-3′ (reverse). Reverse transcription of M. genitalium 23S rRNA was conducted at 55°C for 15 min, followed by 94°C for 2 min. Cycling conditions for both first- and second-round PCRs were as follows: 94°C for 15 s, 62°C for 30 s, and 68°C for 30 s for 40 cycles, followed by 68°C for 5 min. Sanger sequencing was performed with PCR amplicons, using the second-round PCR primers as the sequencing primers.

### Statistical analyses.

For all sexually transmitted organisms (STOs), subjects were considered infected if they had one or more specimens with positive results. The prevalence of infection was calculated using the infected status standard. All tests for 95% confidence intervals (CIs) were two-tailed and performed at the 0.05 significance level, using the efficient score method. Calculation of odds ratios (ORs) and tests for significance were performed as described previously (19), with *P* values of <0.05 being considered significant.

## RESULTS

### Prevalence of M. genitalium infections.

The prevalence rates of M. genitalium infections are presented in Table 1, categorized by gender, age group, symptomatic status, race/ethnicity, and enrollment site. The prevalence of M. genitalium infections in all females (14 to 70 years of age) was 16.3% (95% CI, 13.4 to 19.8%) and that in all males (18 to 78 years of age) was 17.2% (95% CI, 13.9 to 21.0%). M. genitalium infections were significantly more prevalent in subjects ≤30 years of age than in subjects >30 years of age (female, 19.2% versus 7.2% [OR, 2.67; *P* = 0.0075]; male, 22% versus 9.2% [OR, 2.39; *P* = 0.0043]). The prevalence of M. genitalium infections in females was highest in the youngest age groups (30.0% for ages 14 to 17 years and 25.0% for ages 18 to 20 years) and declined in older age groups (13.3% for ages 21 to 30 years, 9.6% for ages 31 to 40 years, 2.9% for ages 41 to 50 years, 6.3% for ages 51 to 60 years, and 0% for ages 61 to 70 years). In males, the prevalence of M. genitalium infections was 12.5% for ages 18 to 20 years, peaked at ages 21 to 30 years (24.1%), and declined thereafter (14.7% for ages 31 to 40 years, 6.1% for ages 41 to 50 years, 3.4% for ages 51 to 60 years, and 0% for ages 61 to 78 years). Subjects self-identifying as black had the highest prevalence of M. genitalium infections (female, 23.5%; male, 27.9%). The highest rates of M. genitalium infections in female subjects occurred among those enrolled from a midwestern adolescent gynecology clinic (28.9%) and a mid-Atlantic public health clinic (20.5%). Male subjects enrolled from a mid-Atlantic public health clinic and a midwestern hospital-based high-risk STD clinic system had the highest M. genitalium infection rates (25.3% and 50%, respectively), although male enrollment at the latter site was low (*n* = 6). Symptomatic subjects had higher prevalence rates of M. genitalium infections (symptomatic versus asymptomatic: female, 21.1% versus 7.5%; male, 19.3% versus 15.4%). Thus, in addition to age differences, significant risk factors for M. genitalium infections were black race (female black versus white: OR, 4.79 [*P* < 0.0001]; male black versus white: OR, 6.54 [*P* < 0.0001]), non-Hispanic ethnicity (female non-Hispanic versus Hispanic: OR, 9.41 [*P* = 0.0276]; male non-Hispanic versus Hispanic: OR, 4.02 [*P* = 0.0089]), and symptomatic status in females (versus asymptomatic status: OR, 2.83 [*P* < 0.0007]).

Rates of detection of M. genitalium and other sexually transmitted organisms (C. trachomatis, N. gonorrhoeae, and T. vaginalis) for both female and male subjects are presented in Table 2. Based on the infected status standard, the overall female M. genitalium prevalence rate of 16.3% was significantly higher than the prevalence rates for C. trachomatis (9.3% [OR, 1.75; *P* = 0.0035]) and N. gonorrhoeae (1.9% [OR, 8.4; *P* < 0.0001]). For males, the prevalence rate for M. genitalium (17.2%) was significantly higher than those for N. gonorrhoeae (4.2% [OR, 4.11; *P* < 0.0001]) and T. vaginalis (5.6% [OR, 3.08; *P* < 0.0001]) but not significantly different from that for C. trachomatis (17.8% [OR, 0.961; *P* = 0.8218]).

**TABLE 2:** Prevalence of *M. genitalium*, C. trachomatis, N. gonorrhoeae, and T. vaginalis detected in 515 females and 431 males

Organism	Infected (% [95% CI])	
	Female	Male
M. genitalium	16.3 (13.4–19.8)^*a*^	17.2 (13.9–21)^*b*^
C. trachomatis	9.3 (7.1–12.1)	17.8 (14.5–21.8)
N. gonorrhoeae	1.9 (1.1–3.5)	4.2 (2.7–6.5)
T. vaginalis	25.2 (21.7–29.2)	5.6 (3.8–8.2)

a OR of 1.75 (*P* = 0.0035) versus *C. trachomatis*, OR of 8.4 (*P* < 0.0001) versus N. gonorrhoeae, and OR of 0.646 (*P* < 0.0044) versus T. vaginalis.

b OR of 4.11 (*P* < 0.0001) versus *N. gonorrhoeae* and OR of 3.08 (*P* < 0.0001) versus T. vaginalis.

### Rates of single infections and coinfections of M. genitalium with C. trachomatis, N. gonorrhoeae, and T. vaginalis.

**FIG 1:**
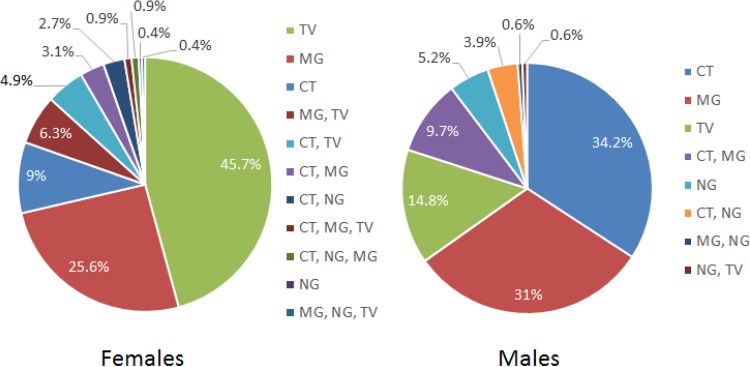
Distribution of single-infection and coinfection rates of sexually transmitted organisms among 223 women and 155 men who tested positive for a sexually transmitted infection. TV, Trichomonas vaginalis; MG, Mycoplasma genitalium; CT, Chlamydia trachomatis; NG, Neisseria gonorrhoeae.

Fig. 1 shows the distribution of single infections and coinfections of the four STOs tested for, among 233 female and 155 male subjects positive for one or more STOs. The majority of subjects were infected with a single organism (female, 80.7%; male, 85.2%). The most prevalent single infection in females was T. vaginalis (45.7% of all female subjects with a STI), followed by M. genitalium (25.6%), C. trachomatis (9%), and N. gonorrhoeae (0.4%). Single M. genitalium infections represented 68.7% of all M. genitalium infections in female subjects. By comparison, 71.6% of C. trachomatis infections in female subjects were single, as were 78.5% of all T. vaginalis infections. M. genitalium presented in dual infections with T. vaginalis and C. trachomatis in 6.3% and 3.1% of infected subjects, respectively. Infections with combinations of three or four organisms were rare (<1% per combination).

Among male subjects, the most prevalent single infection was C. trachomatis (34.2% of all male subjects with a STI), followed by M. genitalium (31%) and N. gonorrhoeae (5.2%). In male subjects, single M. genitalium infections represented 75% of all M. genitalium infections, single C. trachomatis infections represented 41.7% of all C. trachomatis infections, and single T. vaginalis infections represented 95.8% of all T. vaginalis infections. The most prominent dual infection combination among males with a STI was M. genitalium and C. trachomatis (9.7%); other dual infection combinations together represented 5.1% of infected subjects. There were no higher-order infection combinations in males.

### Rates of macrolide antibiotic resistance markers.

Reverse transcription-PCR and Sanger sequencing of M. genitalium 23S rRNA was used to assess the rates of macrolide resistance markers in a subset of subjects in the study (Table 3). Overall, 65 of 128 female subjects (50.8% [95% CI, 42.2 to 59.3%]) and 21 of 50 male subjects (42% [95% CI, 29.4 to 55.8]) harbored M. genitalium organisms with base position 2058/2059 mutations in 23S rRNA. Among both male and female subjects, the macrolide resistance marker prevalence was slightly higher in younger (14 to 24 years of age) versus older (25 to 47 years of age) subjects and higher in black versus white subjects, although the differences were not statistically significant. Non-Hispanic females had a >3-fold higher 23S rRNA mutation frequency than did Hispanic females (54.5% versus 16.7%), although the number of Hispanic female specimens evaluated by sequencing was small (*n* = 6).

**TABLE 3:** M. genitalium macrolide antibiotic resistance marker frequency in female and male subjects

Category	No. with 23S rRNA 2058/2059^*a*^ mutation/total no. (% [95% CI])	
	Female	Male
All subjects	65/128 (50.8 [42.2–59.3])	21/50 (42 [29.4–55.8])
Race/ethnicity		
Black	59/102 (57.8 [48.1–67])^*b*^	20/44 (45.4 [31.7–60])
White	5/22 (22.7 [10.1–43.4])	1/4 (25 [4.6–70])
Unknown race	1/4 (25 [4.6–70])	0/2 (0 [0–65.8])
Hispanic	1/6 (16.7 [30.1–56.4])	1/2 (50 [9.5–90.6])
Non-Hispanic	61/112 (54.5 [45.2–63.4])	18/43 (41.8 [28.4–56.7])
Unknown ethnicity	3/10 (30 [10.8–60.3])	2/5 (40 [11.8–76.9])
Symptomatic status		
Symptomatic	57/102 (55.9 [46.2–65.1])^*c*^	9/21 (42.9 [24.5–63.5])
Asymptomatic	8/26 (30.8 [16.5–50])	12/29 (41.4 [25.5–59.3])
Age		
14–24 yr	52/97 (53.6 [43.7–63.2])	12/24 (50 [31.4–68.6])
25–47 yr	13/31 (41.9 [26.4–59.2])	9/26 (34.6 [19.4–53.8])

a E. coli 23S rRNA numbering.

b OR of 2.54 (*P* = 0.0734) versus white females.

c OR of 1.81 (*P* = 0.172) versus asymptomatic females.

## DISCUSSION

This study investigated the prevalence of M. genitalium and three other sexually transmitted organisms in a diverse cohort of female and male subjects seeking care at a variety of health care facilities in the United States. To our knowledge, these data represent the broadest geographic population studied to date for ascertainment of the M. genitalium prevalence in the United States. The results show high rates of M. genitalium infections in adolescent and adult women and men, with prevalence rates exceeding 24% in young adult men and 25% in adolescent and young adult women and then gradually decreasing at older age for both genders. M. genitalium prevalence rates were also elevated for female and male subjects who self-identified as black or of non-Hispanic ethnicity and for subjects who sought care at public health, family planning, adolescent gynecology, or STD health care facilities in the Midwest, Mid-Atlantic, or Southeast regions of the United States. Subjects who received care at family medicine or OB-GYN practices in the Northeast or at a public health clinic in the Southwest had low rates of M. genitalium infection, as did subjects who self-identified as being white or Asian or of Hispanic ethnicity. Hispanic females especially appeared to harbor a low burden of M. genitalium infection, with only 1 subject testing positive of 49 evaluated.

The overall prevalence rates for M. genitalium infections reported here are similar to those reported previously in smaller studies of high-risk populations in the United States (6, 9, 10, 14, 15, 20) and in the European community (21, 22), Australia and New Zealand (23–25), Asia (26, 27), and Africa (28, 29). Age-related trends for M. genitalium infection prevalence similar to those reported here have also been observed previously (7, 30), and the low prevalence (∼1.0%) of M. genitalium infections observed here among subjects attending family medicine or OB-GYN facilities is on par with that reported previously for low-risk segments of the general population in the United States (8). In addition, the higher rates of M. genitalium infections seen in black subjects are in agreement with previous studies involving at-risk African Americans in the United States (14, 15, 31). These new results reinforce the need for effective STI detection, treatment, and education efforts among young African Americans engaging in high-risk sexual practices.

Our data differ from those reported previously (14, 32) in that testing for M. genitalium and other sexually transmitted organisms using sensitive NAAT methods revealed that the majority of subjects (>80%) of both genders were infected with a single organism and infections with ≥2 organisms were relatively uncommon. The reason for this difference is not clear, although the use of geographically and clinically diverse medical practices for the initiation of patient encounters in this study might have revealed infection patterns not apparent in previous studies with smaller or more geographically constrained enrollments. Regardless of the reason, we found here that, for M. genitalium, two-thirds of females and three-quarters of males who tested positive for the organism by a NAAT did not have a concurrent infection with another STO. Similarly, for C. trachomatis, approximately one-half of males and three-quarters of females who were positive for the organism by a NAAT had a single infection; for T. vaginalis, three-quarters of females and nearly all males had single infections. This apparent predominance of subjects harboring single-organism infections has implications for the diagnosis and treatment of individuals who are at high risk for STIs. Current treatment guidelines recommend antibiotic therapy that is specific to each microorganism (33) and, since coinfection with one or more STOs may be uncommon, the choice of therapy for managing mucosal epithelial inflammatory conditions should be guided by the employment of clinically validated diagnostic methods, rather than a syndromic management approach.

For M. genitalium infections, macrolide antibiotics are the current treatment of choice (33). In recent years, numerous studies on cohorts originating outside the United States have reported high detection rates for a macrolide antibiotic resistance genotype of M. genitalium, thereby limiting the options for treatment (18, 34–37). In the present study, we also found high rates of a M. genitalium macrolide resistance phenotype (51% in females and 42% in males), indicating that resistance to this class of drugs is not a regional phenomenon outside North America but is likely to be endemic in the U.S. population as well. Confirmation of this finding using broader evaluations of the U.S. population would complicate current treatment methods for M. genitalium infections and might indicate a need for comprehensive reassessment of current diagnostic and pharmacological solutions for treating infected patients.

Major strengths of this study include the number of subjects evaluated (*n* = 946), the demographic and geographic diversity of the population sample studied, and the variety of clinical practice types employed for enrollment of subjects seeking care. Other important attributes of the study were the use of sensitive NAATs to detect all four sexually transmitted organisms and the use of RNA sequencing to detect expression of the M. genitalium macrolide resistance phenotype.

Interpretation of our results is constrained by several limitations. First, subjects of Asian descent or Hispanic ethnicity constituted relatively small proportions of all enrolled subjects (0.7% and 13.1%, respectively). Therefore, the findings shown here may not be representative of the true STI burden in these populations in the United States. Second, the TMA assay used for M. genitalium detection is a research-use-only assay that has not been subjected to formal clinical validation. In the absence of confirmatory testing by second and third sensitive NAATs, the M. genitalium results reported include unknown numbers of false-positive and false-negative results. However, this concern is mitigated by previous studies showing good clinical agreement between the TMA NAAT used and PCR-based M. genitalium NAATs (5, 6). Third, only a subset of specimens from enrolled subjects were available for testing for macrolide resistance makers, thus introducing a selection bias of unknown magnitude to the final prevalence estimates for the resistance phenotype in the cohort. Finally, our assessment of macrolide antibiotic resistance relied solely on detection of the A2058/2059 mutations that are known to result in a high-level macrolide resistance phenotype (18). We did not investigate markers for other possible mechanisms conferring macrolide resistance, such as the *mtr* promoter mutations found in macrolide-resistant N. gonorrhoeae (38). Hence, the macrolide resistance prevalence estimates reported here may underestimate the true prevalence of resistance to this antibiotic class in M. genitalium in the U.S. population.

The results of this study show a high prevalence of single M. genitalium infections and limited coinfection with other sexually transmitted organisms in a broad cross section of U.S. subjects at risk of acquiring a sexually transmitted infection. These findings support the monitoring of M. genitalium infections in high-risk populations as part of public health STI screening programs. The high rate of macrolide antibiotic resistance markers found in this study is concordant with resistance rates seen in other populations throughout the world and supports the testing of *M. genitalium*-positive subjects for macrolide resistance before the initiation of treatment.
